# Molecular characterization of *Streptococcus agalactiae* and *Streptococcus dysgalactiae* causing bovine mastitis in the southern region of Bangladesh

**DOI:** 10.5455/javar.2023.j667

**Published:** 2023-06-30

**Authors:** Zinat Farzana, Ayan Saha, AMAM Zonaed Siddiki

**Affiliations:** 1 Department of Genetic Engineering and Biotechnology, East West University, Dhaka, Bangladesh; 2 Department of Bioinformatics and Biotechnology, Asian University for Women, Chattogram, Bangladesh; 3 Department of Pathology & Parasitology, Chittagong Veterinary and Animal Sciences University, Chattogram, Bangladesh

**Keywords:** Bovine mastitis, Streptococcus agalactiae, Streptococcus dysgalactiae, PCR, sequencing, phylogenetic analyses

## Abstract

**Objective:**

This study was conducted to validate polymerase chain reaction (PCR) as a confirmatory diagnostic tool to find out the presence and frequency of *Streptococcus agalactiae* (S. agalactiae) and *Streptococcus dysgalactiae* (*S. dysgalactiae*) in mastitic milk samples obtained from dairy cows in the southern region of Bangladesh.

**Materials and Methods:**

A total of 196 samples of bovine milk were collected from various dairy farms in the Chattogram metropolitan area of the southern part of Bangladesh. DNA extracted from isolates obtained by culturing California mastitis test (CMT)-positive mastitic milk samples (*n =* 146) on 5% sheep blood agar was used as a template for PCR. Two sets of specific primers based on the *16S rRNA* gene were used to discriminate between *S. agalactiae* and *S. dysgalactiae*. Four PCR products were subjected to sequencing, followed by phylogenetic analysis.

**Results:**

The PCR analyses revealed that out of the 146 CMT-positive milk samples tested, 29 samples were positive for* S. agalactiae* (19.86%), while 26 samples were positive for *S. dysgalactiae* (17.81%). Further sequence analysis of the corresponding PCR products and bioinformatics analysis verified the results.

**Conclusion:**

The study proves the efficiency of PCR as a useful diagnostic approach to determine the presence and prevalence of* S. agalactiae *and* S. dysgalactiae* in mastitic milk samples obtained from dairy cows.

## Introduction

Bovine mastitis (BM), one of the costliest diseases of the dairy industry, causes huge economic losses worldwide, mainly due to the reduction in milk yield, decrease in milk quality, and increased production costs. It results from the release of leukocytes into the mammary glands, generally in response to an invasion of bacteria into the teat canal. Nearly any bacterial or mycotic organism that has the capacity to opportunistically invade tissue and cause infection can cause mastitis. Nevertheless, different species of streptococci, staphylococci, and coliforms cause the most infections [1]. The bacteria causing BM can be classified as environmental or contagious based on their primary reservoir (environment *vs.* infected mammary gland quarter). The main environmental bacteria include *Escherichia coli*, *Streptococcus dysgalactiae*, *Streptococcus parauberis*, and *Streptococcus uberis*. *Staphylococcus aureus*, and *Streptococcus agalactiae* are the main contagious bacteria [2,3].

Different pathogenic bacteria can be transmitted from mastitis-diseased cattle to humans through milk consumption, which cause different diseases in humans, including tuberculosis, brucellosis, diphtheria, streptococcal sore throat, scarlet fever, Q-fever, etc. Despite pasteurization techniques, these diseases, illnesses, and disease outbreaks are caused by several bacteria. *Streptococcus agalactiae*, one of the foremost causes of BM with important economic implications for the dairy cattle industry worldwide, is also a significant human pathogen that can cause infections in neonates, pregnant women, and the elderly. *Streptococcus dysgalactiae* is also one of the most prevalent pathogens causing BM globally, which results in financial damage and decreased animal welfare. It also has the potential to infect humans. Therefore, control and prevention of BM caused by* S. agalactiae* and *S. dysgalactiae* will increase the quantity and quality of milk yield and lead to enhanced animal health and public wellbeing [4,5].

Intramammary infections are often described as subclinical or clinical mastitis. Though the infection is present, there are no apparent signs of local inflammation or systemic involvement in subclinical mastitis (SCM) [6]. Clinical mastitis is a type of udder infection that usually appears suddenly, causing redness and swelling in the udder, along with fever and changes in milk quality. In contrast, SCM is a less severe form of infection that often goes unnoticed, occurring 15 to 40 times more frequently than clinical mastitis. SCM can last longer than the clinical form and may have more subtle symptoms [7]. If SCM is not diagnosed in its initial stage, it may lead to clinical mastitis, which is incurable in most cases. In addition, the whole herd of ruminants is at constant risk of infection if SCM remains undetected. Therefore, early detection of the occurrence of the pathogens causing SCM is of enormous significance to prevent the disease effectively, guide treatment, and save human consumers from the associated diseases resulting from the ingestion of contaminated milk from cows with mastitis [8,9].

In most clinical laboratories, bacterial identification is done by microbiological culturing of milk, followed by biochemical tests of the bacteria isolated. The identification of microorganisms in milk or in other organic samples through microbial culturing requires the step of growing the bacteria in culture media and is, therefore, time-consuming [10]. Besides this, the identification of microorganisms by observing the phenotypic characteristics of cultured bacteria occasionally provides bewildering results. On the other hand, fast identification approaches, specifically polymerase chain reaction (PCR)-based techniques, have the potential to be exceedingly specific and can also distinguish between closely related organisms, like *S. agalactiae* and *S. dysgalactiae* [11–13]. Therefore, the objective of this study is to validate PCR as a confirmatory diagnostic tool to identify *S. agalactiae *and *S. dysgalactiae* obtained by culturing mastitic milk samples on 5% sheep blood agar, which is a selective medium for these species. Because knowledge about the prevalence of *S. agalactiae* and *S. dysgalactiae* in dairy cows is limited in the southern region of Bangladesh, the study also aims to determine the prevalence of these two species in this area, which will help different government and non-government aid organizations to improve dairy productivity.

## Materials and Methods

### Collection of milk samples and California mastitis test (CMT)

A total of 196 samples of bovine milk were collected under aseptic conditions from various dairy farms in the Chattogram metropolitan area of southern Bangladesh. A commercial CMT kit (Leucocytest^®^, Synbiotics Corporation-2, Alexander Fleming-69007 Lyon, France) was used to determine SCM. Before sampling, cotton was soaked in 70% ethanol, and then the teat end was scrubbed with it. The CMT paddle was washed every time with distilled water and then cleaned with 70% ethanol before use. Two milliliters of milk from each quarter of a cow’s udder were taken in each of the four cups of the CMT paddle. Following the addition of an equal volume of CMT reagent to each cup, the paddle was rotated in a circular motion to thoroughly mix the contents and scored visually within 20 sec, depending on the gel formed. If no gel was formed within 20 sec, the test result was taken as negative. In the field, all CMT-positive milk samples were collected into sterilized tubes, followed by freezing at −20°C. The CMT test result is shown in Table 1.

### Bacteriological methods

After thawing, 10 μl from each of the milk samples was streaked onto a blood agar base with 5% sheep blood (Oxoid, UK) in a Petri plate. Incubation was done for 24 h at 37°C. The bacteria were identified by standard laboratory methods [14]. Bacterial isolates were recognized as streptococci if they were Gram-positive cocci with a negative catalase reaction. Streptococci were detected as *S. agalactiae *based on a positive Christie–Atkins–Munch-Peterson (CAMP) test and a negative esculin hydrolysis test. Streptococci were found to be *S. dysgalactiae *according to a negative CAMP test and a negative esculin hydrolysis test [15].

**Table 1.** Screening result by CMT test.

Sources (Dairy farms)	Total no. of samples	No. of CMT-positive samples	No. of CMT-negative samples	Percentage of CMT-positive samples (%)
A	110	80	30	72.73
B	16	12	4	75
C	28	19	9	67.86
D	42	35	7	83.33
Total	196	146	50	74.49

*Here A, B, C, D denotes the names of different dairy farms from where milk was collected.

### DNA extraction from milk

DNA was extracted from the isolates obtained by bacteriological culture from CMT-positive milk samples for amplification through PCR. DNA was extracted by the sodium dodecyl sulfate (SDS)-phenol–chloroform–isoamyl alcohol method [16] with some modifications. In the nutrient broth medium, a pure bacterial culture from sheep blood agar was sub-cultured. 1.5 ml of inoculated broth was taken into a microcentrifuge and centrifuged for 5 min. Then the supernatant was thrown away. Pellet was resuspended in 600 μl NTE buffer [0.1 M NaCl, 20 mM Tris-HCl (pH 7.4), and 1 mM EDTA (pH 7.5)] containing SDS and proteinase-K to concentrations of 0.5% and 100 μg, respectively. The suspension was thoroughly mixed, followed by incubation at 56°C for 1 h. In this suspension, 600 μl of phenol–chloroform–isoamylalcohol (25:24:1) (volume:volume) was added, followed by centrifugation at 10,000×*g* for 5 min. The upper viscous supernatant was removed in a fresh 2.0 μl centrifuge tube. The process was repeated until the white interface was lost. The upper aqueous phase was then re-extracted with an equal volume of chloroform–isoamyl alcohol (24:1) (volume:volume). After the collection of the resulting aqueous phase, the one-tenth volume of 3 M sodium acetate (pH 5.2) and two volumes of chilled 100% ethanol was mixed with it. After mixing the solution thoroughly, DNA precipitation was done by keeping it at −20°C for 45 min. 70% ethanol was used to wash pelleted DNA after centrifugation at 10,000×*g* for 15 min at 4°C. Then, the DNA pellet was suspended in TE buffer [10 mM Tris HCl, 5 mM EDTA (pH 8.0)] [17].

### Oligonucleotide primers

For detecting *S. agalactiae* and* S. dysgalactiae*, two sets of oligonucleotide primers were used based on the *16S rRNA *gene, as described by Riffon et al. [3] in 2001. Specific primers were used, resulting in product sizes of 405 and 281 bp for* S. agalactiae* and *S. dysgalactiae*, respectively. The primers used in this research are listed in Table 2.

### PCR amplification 

The extracted DNA was subjected to PCR analysis based on the *16S rRNA* gene. The required PCR reaction materials, which include Go* Taq* Green Master Mix (2×) (Promega), primers (forward and reverse), a template, and nuclease-free water, were taken in a PCR tube, mixed thoroughly, and then the run conditions were set as primary denaturation at 94°C for 2 min, denaturation at 94°C for 45 sec, annealing (at 60°C for *S. agalactiae* and at 57°C for* S. dysgalactiae*) for 1 min, and extension at 72°C for 2 min. A total of 35 PCR cycles were performed in a thermocycler (Bio-Rad, USA), and the preparation was kept at 72°C for 10 min after the final cycle to complete the reaction. The PCR products were kept at 4°C in the thermocycler before collection [13].

### Analysis of PCR products

**Figure 1.**
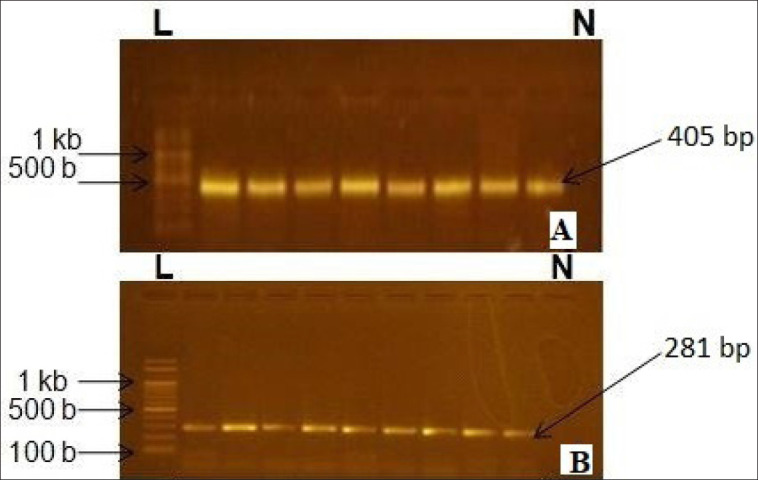
(A) Band of *S. agalactiae* in 2% agarose gel (B) Band of *S. dysgalactiae* in 2% agarose gel.

Electrophoresis of PCR products was conducted on a 2% agarose gel containing 0.2 μg/ml ethidium bromide in Tris borate EDTA electrophoresis buffer with a 1,000 bp ladder as a marker for 1 h at 6.5 V/cm. PCR products were quantified by the absorbance of UV at 260 and 280 nm to authenticate their presence and quantity in PCR. Products were visualized by ultraviolet light transillumination (Fig. 1).

### DNA sequencing and submission 

Four PCR products, two from *S. agalactiae* and another two from *S. dysgalactiae*, were subjected to sequencing. Following the sequencing of representative PCR products, the sequences obtained were analyzed by Blast search and submitted to the National Center for Biotechnology Information (NCBI) GenBank database. The search parameters were database: standard database [nucleotide collection (nr/nt)], and optimization: highly similar sequence. The nucleotide sequence data obtained in this research is available in GenBank under accession numbers KF055832, KF055833, KF055834, and KF055835.

### Construction of phylogenetic tree

A phylogenetic tree was built using *16S rRNA* gene sequences from mastitic milk samples of *S. agalactiae* and *S. dysgalactiae* (accession numbers KF055832, KF055833, KF055834, and KF055835) and *16S rRNA* gene sequences from other strains of these two species available in GenBank. During this study, 20 of the top 50 BLAST-derived hits with a significant *E*-value of the *16S rRNA* gene sequences of KF055832, KF055833, KF055834, and KF055835 were retrieved. The sequences were retrieved from the nucleotide database (https://www.ncbi.nlm.nih.gov/nuccore/) and aligned using the multiple alignment program MUSCLE under the default conditions available in MEGA11 [18]. The aligned sequences were trimmed to remove the excess regions flanking both sides of the sequences. Then evolutionary analyses were performed using the maximum likelihood method and the Kimura-2 parameter model in MEGA11 [19]. The test of phylogeny was done using the Bootstrap method by resampling the dataset 1,000 times [20].

**Table 2.** Primer sequence of *S. agalactiae* and *S. dysgalactiae*.

Name of bacteria	Primer name	Sequence of primer
*Streptococcus agalactiae*	Forward primer: 40 Reverse primer: 445	5'-CGC TGA GGT TTG GTG TTT ACA-3' 5'-CAC TCC TAC CAA CGT TCT TC-3'
*Streptococcus dysgalactiae*	Forward primer*:*105 Reverse primer*: *386	5'-AAA GGT GCA ACT GCA TCA CTA-3' 5'-GTC ACA TGG TGG ATT TTC CA-3'

## Results

Totaling 74.49% of the milk samples were found to be associated with mastitis according to the CMT screening presented in Table 1. *Streptococcus agalactiae* and *S. dysgalactiae* were primarily identified from CMT-positive milk samples by culturing the samples on 5% sheep blood agar and by subjecting the isolated bacterial colonies from sheep blood agar to gram staining, the catalase test, the CAMP test, and the esculin hydrolysis test. Culture-derived colonies from 55 samples were found to be Gram-positive with a negative catalase reaction and identified as Streptococci. Isolates from 29 samples were identified as *S. agalactiae* on the basis of a positive CAMP test and a negative esculin hydrolysis test, and isolates from 26 samples were identified as *S. dysgalactiae* on the basis of a negative CAMP test and a negative esculin hydrolysis test (Table 3).

To identify the bacterial strains present, culture-grown colonies were subjected to PCR analysis. By demonstrating positive DNA amplification from bacteria isolated from BM while the closest phylogenetic relative did not, the specificity of the primer pairs used for PCR was established. Negative controls for each bacterial strain were used to regulate the assay. According to Table 2 and Figure 1, PCR amplification using the right primers produced the anticipated DNA fragments, which ranged in size from 94 to 1,318 bp. 29 samples of *S. agalactiae *and 26 samples of *S. dysgalactiae* were found, according to PCR analysis. After PCR amplification, four products in total, two from each of these two species, were subjected to sequencing. The sequence of the amplified DNA was analyzed by BLAST search. 95 out of 101 hits showed about 98% similarity of the *16S rRNA* gene of the isolate identified as *S. agalactiae* in PCR (accession no. KF055832) with different *S. agalactiae* strains (such as accession no. MT626756) in the NCBI standard database. Accession no. KF055833 showed about 95% similarity with *S. agalactiae *(Accession no. MT626756). Accession numbers KF055834 and KF055835 showed about 94% and 97% similarity, respectively, to *S. dysgalactiae* strains with accession numbers MH119693 and NR_027517.

**Figure 2.**
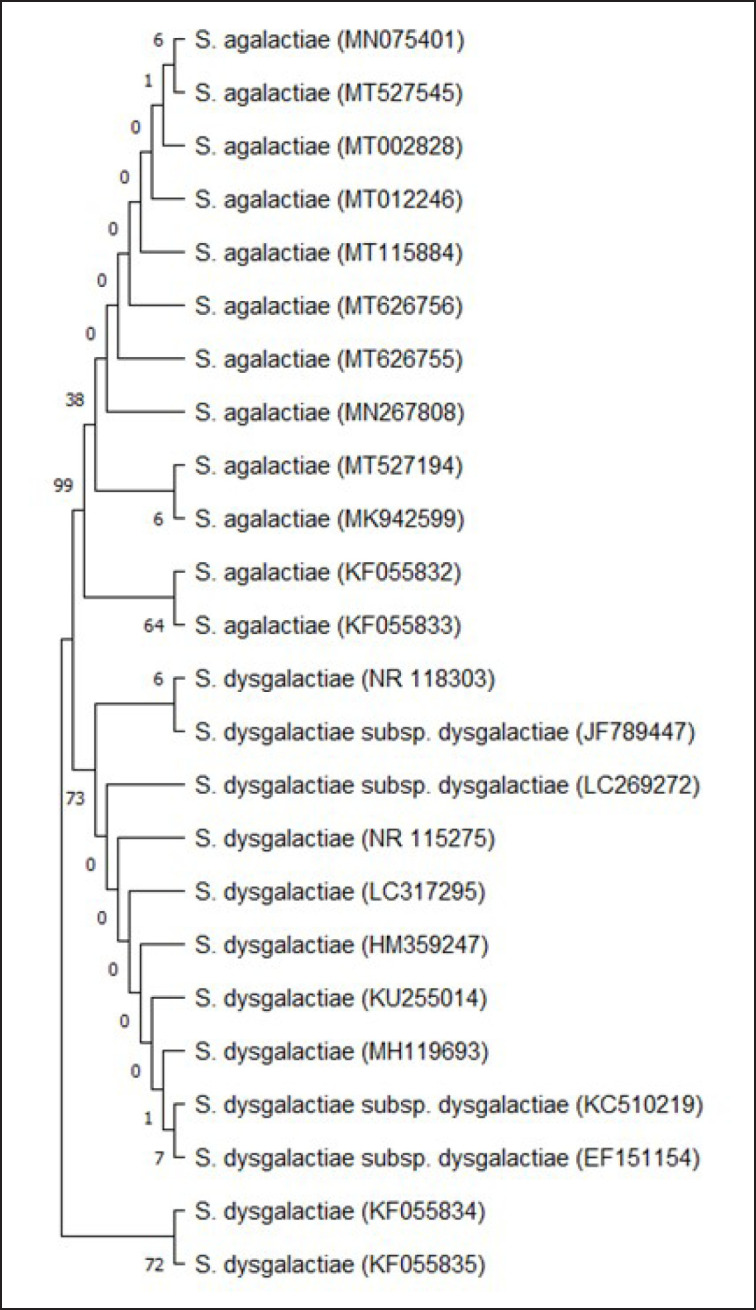
The evolutionary relationships of *S. agalactiae* and *S. dysgalactiae* inferred from *16S rRNA* gene sequences using the maximum likelihood method and Kimura 2-parameter model with 1,000 bootstrap replicates in MEGA11.

An intragenotyping variation of *16S rRNA* gene sequences KF055832, KF055833, KF055834, and KF055835 has been shown in the dendrogram tree (Fig. 2). KF055834 and KF055835 obtained from mastitic milk samples were in the same clade, originating from a common ancestor, as supported by a bootstrap value of 72. All the *S. dysgalactiae* strains tested in this study, except KF055834 and KF055835, originated from a common ancestor, supported by a bootstrap value of 73. The node in the phylogenetic tree with a bootstrap value of 99 suggests that all the *S. agalactiae* tested in this research have emerged from the same origin. The clustering of our sequences KF055832 and KF055833 in the same clade, as well as KF055834 and KF055835 in the same clade, suggests that these strains originated from *Streptococcus* sp. found in Bangladeshi dairy farms. The other *S. agalactiae *and *S. dysgalactiae* strains considered in this study might have originated from different sources found in other countries. As KF055834 has the most genetic distance from other *S. dysgalactiae *sequences, all the other *S. dysgalactiae* sequences might be descended from KF055834. Pairwise genetic distances among all the strains of *S. agalactiae* tested in this study ranged from 0.004 to 0.041, which indicates the close proximity of these strains during evolution. Based on the lower genetic distances observed, we can conclude that the rate of polymorphism among the strains of* S. agalactiae *is not very high. Gene flow might occur during the evolution of *Streptococcus*, causing the generation of different haplotypes in animals and humans from different countries. Therefore, to discriminate between different species of *S. agalactiae*, a complete sequence of the *16S rRNA* gene could be a better characterization tool. The overall mean distance among all the species tested in this study was 0.02.

**Table 3.** Results of different biochemical and bacteriological tests of milk samples.

Total milk samples	CMT test (positive)	5% sheep blood agar test (positive)	Gram staining (positive)	Catalase test (negative)	CAMP test (positive)	Esculin hydrolysis test (negative)
196	146	55	55	55	29	55

## Discussion

Prevention is preferable to treatment, and in the absence of an effective vaccine against BM, controlling the condition is of utmost importance in the dairy industry. To control BM, rapid and specific tests are needed to identify the main bacteria responsible for significant losses in the dairy industry. Traditional methods for identifying BM pathogens are time-consuming, and most commercial identification systems are not designed to detect important veterinary pathogens [13]. Our objective was to develop a detection and identification test for BM pathogens that provides rapid results, requires minimal bacteriological and biochemical testing, and is as specific and cost-effective as possible. Molecular techniques are efficient tools for developing improved diagnostic tests. While laborious techniques like ribotyping exist, new PCR-based methods have been successfully employed to identify many bacteria. The significant advantages of PCR are the ability to use only nanograms of nucleic acid samples, the elimination of extensive culture steps and biochemical tests, speed, and ease of analysis. Using specific primers, PCR is an effective method for the molecular identification of any bacteria. The specificity of the primers described in this article (Table 2 and Fig. 1) was demonstrated by the observation of a single band for each set of primers on agarose gel and the absence of a signal with negative controls. Primers for *S. agalactiae* and *S. dysgalactiae* were designed using 16S rRNA-encoding DNA. Negative controls utilizing the closest phylogenic bacteria discovered in BM confirmed the specificity of each primer set.

In this study, 74.49% of the total milk samples were found to be infected with SCM according to the CMT test, which is higher than the results obtained by Bhuiyan et al. [21], Yimam et al. [22], and Tiwari et al. [23], who reported 28.75%, 7.65%, and 19.3% SCM with the CMT test in dairy cattle, respectively. However, the prevalence of SCM in dairy cows found in this study (74.49%) is similar to the result obtained by Karimuribo et al. [24], who found a prevalence of 75.9% of SCM with the CMT test in dairy cows. The differences in the prevalence of SCM in different studies could be due to differences in sample size and sampling procedures. Variations may also be due to the geographic location, climate, livestock rearing system, husbandry practices, and hygiene [21–24].

Primarily identified *S. agalactiae *and *S. dysgalactiae, *obtained by culturing CMT-positive milk samples on 5% sheep blood agar and subjecting the isolated bacterial colonies from sheep blood agar to gram staining, the CAMP test, and the esculin hydrolysis test, were subjected to PCR analyses. PCR analyses identified 29 isolates from 29 different milk samples (19.86%) as *S. agalactiae* and 26 isolates from 26 different milk samples (17.81%) as *S. dysgalactiae*. This result is comparable with the findings of Moatamedi et al. [16], who reported a 20% *S. agalactiae* and 12.5% *S. dysgalactiae* infection in CMT-positive milk samples in their study [16]. BM is a complex disease that involves the interactions of multiple factors such as husbandry, environmental conditions, milking procedures, a mastitis-control plan, animal risk factors, climate, etc., and thus the prevalence of this disease caused by different causative pathogens also varies.

Sequencing of the 16S rRNA gene of representative PCR products of *S. agalactiae* and *S. dysgalactiae* and subsequent BLAST searches of these sequences against the NCBI Standard Database [nucleotide collection (nr/nt)] showed high sequence similarity (94%–100%) and confirmed the identity of these species. Furthermore, phylogenetic analysis showed the position of these species in the evolutionary tree.

In this study, 100% of the isolates identified as *S. agalactiae* and *S. dysgalactiae* using bacteriological and biochemical methods were found to provide positive results in PCR analyses, confirming the identity of these species. In addition, the sequencing of four representative PCR products from this study confirmed the identity of these pathogens as* S. agalactiae* and* S. dysgalactiae*, which further strengthens the results of PCR analyses and proves the high specificity of the PCR method in detecting colonies of species derived from selective culture medium. However, sequencing all of the PCR products that tested positive as *S. agalactiae* and *S. dysgalactiae* in this research will further validate the use of PCR as a confirmatory tool to identify these species obtained from milk samples by bacteriological and biochemical methods.

This study suggests that SCM caused by *S. agalactiae* and *S. dysgalactiae *is prevalent in dairy cows in the southern region of Bangladesh. Therefore, regular testing and preventive measures are required to plan to prevent the incidence of BM in this region of Bangladesh. In this regard, PCR can be an efficient confirmatory tool to detect these bacterial pathogens in mastitic milk samples with rapidity and specificity, as evident from this research. However, in the future, PCR can be tested to identify bacterial pathogens directly from milk samples, which will make the detection of infection in milk samples the least time-consuming. We are also planning to perform antibiotic sensitivity tests to know the current status of the antibiotic resistance pattern of these two species against currently available antibiotics, which might drive us to develop alternative and/or combined antibiotic therapy.

## Conclusion

The study revealed a considerable occurrence of *S. agalactiae* and *S. dysgalactiae* among dairy cows in the research area. To control the infection these bacteria cause, it is necessary to develop regular testing and prevention programs. This research describes a PCR-based test for identifying and determining the prevalence of *S. agalactiae* and *S. dysgalactiae* associated with BM. The test was conducted directly on culture-derived isolates of milk samples and was found to be specific. Hence, these PCR tests can be effortlessly implemented in clinical veterinary microbiological laboratories to identify isolates obtained directly from culturing mastitic milk samples in a selective culture medium. Therefore, utilizing PCR can be valuable in promoting the prevention of BM.

## Data Availability

Four *16S rRNA* gene sequences from *S. agalactiae *and* S. dysgalactiae* obtained from this research are available in NCBI GenBank under the accession number KF055832, KF055833, KF055834, and KF055835 (https://www.ncbi.nlm.nih.gov/nuccore/).

## List of Abbreviations

BM, bovine mastitis; CAMP test, Christie–Atkins–Munch-Peterson test; CMT, California Mastitis Test; NCBI, National Center for Biotechnology Information; PCR, polymerase chain reaction; SCM, subclinical mastitis; SDS, sodium dodecyl sulfate.
